# Crystal Structures and Mechanical Properties of Ca_2_C at High Pressure

**DOI:** 10.3390/ma9070570

**Published:** 2016-07-14

**Authors:** Qun Wei, Quan Zhang, Meiguang Zhang

**Affiliations:** 1 School of Physics and Optoelectronic Engineering, Xidian University, Xi’an 710071, China; 2 School of Microelectronics, Xidian University, Xi’an 710071, China; quzhang93@foxmail.com; 3 College of Physics and Optoelectronic Technology, Baoji University of Arts and Sciences, Baoji 721016, China

**Keywords:** pressure-induced phase transition, first-principles calculations, Ca_2_C

## Abstract

Recently, a new high-pressure semiconductor phase of Ca_2_C (space group *Pnma*) was successfully synthesized, it has a low-pressure metallic phase (space group *C*2/*m*). In this paper, a systematic investigation of the pressure-induced phase transition of Ca_2_C is studied on the basis of first-principles calculations. The calculated enthalpy reveals that the phase transition which transforms from *C*2/*m*-Ca_2_C to *Pnma*-Ca_2_C occurs at 7.8 GPa, and it is a first-order phase transition with a volume drop of 26.7%. The calculated elastic constants show that *C*2/*m*-Ca_2_C is mechanically unstable above 6.4 GPa, indicating that the structural phase transition is due to mechanical instability. Both of the two phases exhibit the elastic anisotropy. The semiconductivity of *Pnma*-Ca_2_C and the metallicity of *C*2/*m*-Ca_2_C have been demonstrated by the electronic band structure calculations. The quasi-direct band gap of *Pnma*-Ca_2_C at 0 GPa is 0.86 eV. Furthermore, the detailed analysis of the total and partial density of states is performed to show the specific contribution to the Fermi level.

## 1. Introduction

Hitherto, the pressure-composition (*P-x*) phases of binary systems have gained increasing interest and been extensively researched. Among these predicted compounds, some of them have been successfully synthesized [1,2], but the others still need further experiments to confirm their theoretical predictions [3,4]. For the Ca-C system, there are many works that have been done and obtained remarkable achievements [5,6,7,8,9,10,11,12,13,14,15,16]. Gauzzi et al. [5] found the superconductivity will be enhanced in the intercalated graphite CaC_6_ at high pressure. It performs the structural instability and leads to a structural transition with pressure. Nylen et al. [6] studied the structural behavior of CaC_2_ at high pressure via the first-principles calculations. Their results suggest an irreversible amorphization, corroborating the structural peculiarities of acetylide carbides, which persists at high pressure conditions. Li et al. analyzed the pressure-induced superconductivity of CaC_2_ [7]. They uncovered that it is calcium that contributes to the superconducting behavior, and it is capable of stabilizing carbon *sp*^2^ hybridization at a larger range of pressure. Nourbakhsh et al. [8] investigated the magnetism in CaC ionic compound and observed a perfect Fermi level spin polarization and a half-metallic behavior. 

Recently, Li et al. [9] systematically explored all the stable calcium carbides at pressures from 0 to 100 GPa. This resulted in five newly predicted stable stoichiometries (Ca_5_C_2_, Ca_2_C, Ca_3_C_2_, CaC and Ca_2_C_3_). Using in situ synchrotron powder X-ray diffraction measurements, they successfully synthesized the Ca_2_C and Ca_2_C_3_. The Ca_2_C has two phases: the semiconducting phase *Pnma*-Ca_2_C at high pressure and the metallic metastable phase *C*2/*m*-Ca_2_C at low pressure. The *Pnma*-Ca_2_C exists in the pressure range of 7.5–100 GPa and possesses the isolated C anions. Carbon atoms polymerize to isolated dumbbells, occurring a unique metallic metastable *C*2/*m*-Ca_2_C which provides an example of 2D metal. The metal calcium atom of *C*2/*m*-Ca_2_C develops a negative Bader charge, confronting a more electronegative carbon atom. Due to these intriguing properties, in this paper, we will focus on the Ca_2_C, presenting its structural, elastic and electronic properties, and systematically investigating the pressure-induced phase transition mechanism. The enthalpy is calculated to reveal the phase transition pressure. In addition, the elastic constants, modulus and anisotropy are calculated to study the elastic properties. Meanwhile, the electronic band structures and the total and partial density of states are analyzed.

## 2. Results and Discussion

**Figure 1:**
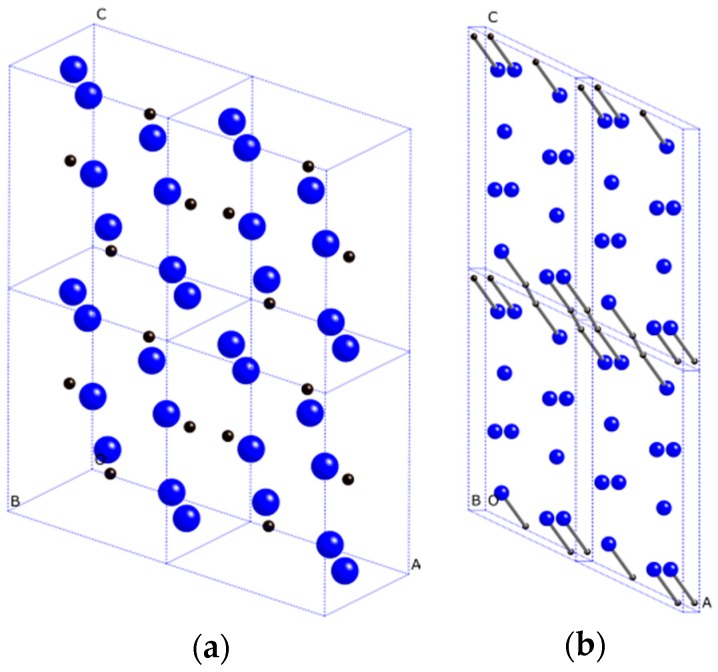
Crystal structures of Ca_2_C. (**a**) *Pnma*-Ca_2_C; (**b**) *C*2/*m*-Ca_2_C. The black and blue spheres represent C and Ca atoms, respectively.

The 2 × 1 × 2 supercell structures of Ca_2_C are illustrated in Figure 1. The black and blue spheres represent C and Ca atoms, respectively. At zero pressure, the optimized lattice parameters of *Pnma*-Ca_2_C are a = 6.677 Å, b = 4.384 Å, c = 7.979 Å with two inequivalent Ca atoms occupying 4c (0.0119, 0.2500, 0.8302), 4c (0.1476, 0.2500, 0.4109) and C atoms occupying 4c (0.2521, 0.2500, 0.0918) Wyckoff positions. For *C*2/*m*-Ca_2_C, the optimized lattice parameters are a = 7.166 Å, b = 3.775 Å, c = 15.490 Å, and β = 122.9°. The Wyckoff positions of *C*2/*m*-Ca_2_C are Ca1: 4i (0.2715, 0.0000, −0.1120), Ca2: 4i (−0.2440, 0.0000, −0.3781) and C: 4i (−1.0479, 0.0000, −0.9705). For *Pnma*-Ca_2_C (see Figure 1a), carbon atoms are isolated anions, whereas the carbon dimers are observed in *C*2/*m*-Ca_2_C (Figure 1b). The interatomic distance of Ca-C for *C*2/*m*-Ca_2_C is 2.44 Å in length, and the C-C bond length is 1.29 Å.

**Figure 2:**
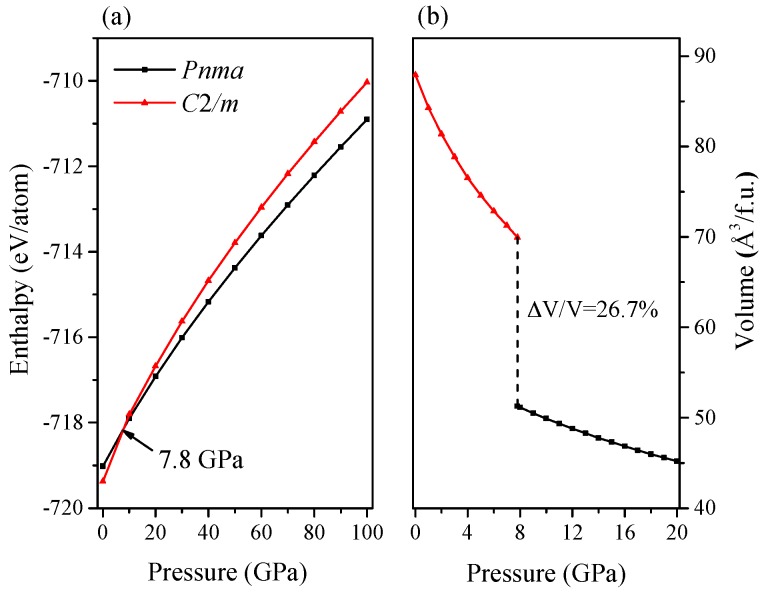
Enthalpy (**a**) and volume (**b**) as a function of pressure. The black and red solid lines represent *Pnma*-Ca_2_C and *C*2/*m*-Ca_2_C, respectively.

To determine the phase transition pressure of Ca_2_C, the enthalpy differences between two structures are plotted as a function of pressure up to 100 GPa in Figure 2a. There is an intersection between the two enthalpy curves, indicating that the *C*2/*m*-Ca_2_C phase transforms to the *Pnma*-Ca_2_C phase at 7.8 GPa and the *Pnma*-Ca_2_C is more stable than the *C*2/*m*-Ca_2_C above this pressure point. The known transition pressure data is 7.5 GPa [9], and it is in a good agreement with our result. Meanwhile, the dependence of volume on pressure is presented in Figure 2b. The *C*2/*m*-Ca_2_C is larger than the *Pnma*-Ca_2_C in volume. The change of volume at 7.8 GPa shows that the phase transition is first-order with a volume drop of 26.7%. To interpret this large volume collapse, we estimated the ionic radii of the C and Ca within these two structures at 7.8 GPa through Bader charge analysis. The obtained results are listed in Table 1. The calculated charges of the two Ca_2_C phase show increasing trends from *C*2/*m*-Ca_2_^0.928^C^−0.928^ to *Pnma*-Ca_2_^2.348^C^−2.348^ at phase transition pressure point. Compared to *C*2/*m*-Ca_2_C phase (*r*_Ca_ = 1.871 Å, *r*_C_ = 1.534 Å), the ionic radius of Ca in the *Pnma*-Ca_2_C phase is much shorter (1.485 Å), whereas the ionic radius of C (1.788 Å) in *Pnma*-Ca_2_C is relatively longer. Since the contribution from Ca atom is much more than that of C atom to the volume of Ca_2_C, the volume collapse from *C*2/*m* to *Pnma* phase is very large.

**Figure 3:**
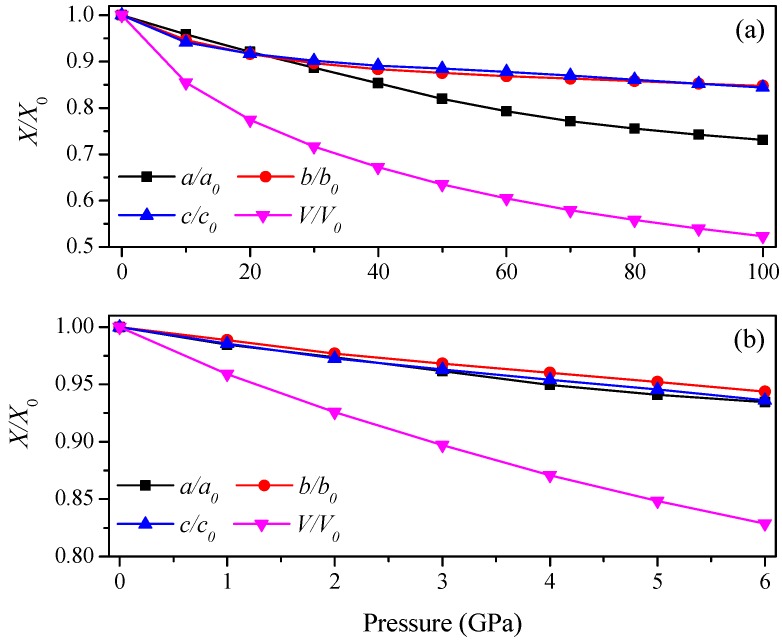
Lattice parameters *X*/*X*_0_ as a function of pressure. (**a**) *Pnma*-Ca_2_C; (**b**) *C*2/*m*-Ca_2_C.

The lattice parameters of Ca_2_C at different pressures are listed in Table 2. In Table 2, an excellent agreement with the previous theoretical and experimental values is shown [9]. The calculated lattice parameters decrease with pressure. To get more details, the variations of lattice parameters *X*/*X*_0_ of the two Ca_2_C phases with pressure are shown in Figure 3. For *Pnma*-Ca_2_C (see Figure 3a), along the *b*- and *c*-axis, the degrees of anti-compression along these two directions are almost the same. At low pressure range (*P* < 23 GPa), the incompressibility along *a*-axis is larger than that along *b*- and *c*-axis, which is contrary to the case at high pressure range (*P* > 23 GPa). In Figure 3b, the changes of lattice parameters along the *a*-, *b*- and *c*-axis are similar for *C*2/*m*-Ca_2_C when below 6 GPa, suggesting the same incompressibility along these three directions.

The calculated elastic constants and moduli of Ca_2_C at 0 GPa and high pressures are shown in Table 3. The strain-stress method was used to calculate the single crystal elastic constants. A small finite strain was applied on the optimized structure and the atomic position was fully optimized. Then, the elastic constants were obtained from the stress of the strained structure. The generalized Born’s mechanical stability criteria of orthorhombic phase at 0 GPa are given by [17,18]:

(1) $$C_{ii}>0,i=1\ldots 6,$$

(2) $$[C_{11}+C_{22}+C_{33}+2(C_{12}+C_{13}+C_{23})]>0,$$

(3) $$(C_{11}+C_{22}-2C_{12})>0,$$

(4) $$(C_{11}+C_{33}-2C_{13})>0,$$

(5) $$(C_{22}+C_{33}-2C_{23})>0$$

The stability criteria of monoclinic phase at 0 GPa are given by [17,18]:

(6) $$C_{ii}>0,i=1\ldots 6,$$

(7) $$[C_{11}+C_{22}+C_{33}+2(C_{12}+C_{13}+C_{23})]>0,$$

(8) $$(C_{33}C_{55}-C_{35}^{2})>0,$$

(9) $$(C_{44}C_{66}-C_{46}^{2})>0,$$

(10) $$(C_{22}+C_{33}-2C_{23})>0,$$

(11) $$[C_{22}(C_{33}C_{55}-C_{35}^{2})+2C_{23}C_{25}C_{35}-C_{23}^{2}C_{55}-C_{25}^{2}C_{33}]>0,$$

(12) $$2[C_{15}C_{25}(C_{33}C_{12}-C_{13}C_{23})+C_{15}C_{35}(C_{22}C_{13}-C_{12}C_{23})+C_{25}C_{35}(C_{11}C_{23}-C_{12}C_{13})]-[C_{15}^{2}(C_{22}C_{33}-C_{23}^{2})+C_{25}^{2}(C_{11}C_{33}-C_{13}^{2})+C_{35}^{2}(C_{11}C_{22}-C_{12}^{2})]+C_{55}g>0,$$

(13) $$g=C_{11}C_{22}C_{33}-C_{11}C_{23}^{2}-C_{22}C_{13}^{2}-C_{33}C_{12}^{2}+2C_{12}C_{13}C_{23}$$

The mechanical stability in crystals under isotropic pressure is provided by Ref. [19]. This requires the symmetric matrix

(14) $$\hat{G}=\left[\begin{array}{cccccc} {\tilde{C}}_{11} & {\tilde{C}}_{12} & {\tilde{C}}_{13} & 2C_{14} & 2C_{15} & 2C_{16}\\ {\tilde{C}}_{21} & {\tilde{C}}_{22} & {\tilde{C}}_{23} & 2C_{24} & 2C_{25} & 2C_{26}\\ {\tilde{C}}_{31} & {\tilde{C}}_{32} & {\tilde{C}}_{33} & 2C_{34} & 2C_{35} & 2C_{36}\\ 2C_{41} & 2C_{42} & 2C_{43} & 4{\tilde{C}}_{44} & 4C_{45} & 4C_{46}\\ 2C_{51} & 2C_{52} & 2C_{53} & 4C_{54} & 4{\tilde{C}}_{55} & 4C_{56}\\ 2C_{61} & 2C_{62} & 2C_{63} & 4C_{64} & 4C_{65} & 4{\tilde{C}}_{66} \end{array}\right]$$

has a positive determinant. In *Ĝ* matrix,

(15) $$\begin{array}{lll} {\tilde{C}}_{\alpha \alpha }=C_{\alpha \alpha }-P, & \alpha =1,2,\ldots ,6 & \\ {\tilde{C}}_{12}=C_{12}+P, & {\tilde{C}}_{13}=C_{13}+P, & {\tilde{C}}_{23}=C_{23}+P \end{array}$$

where *P* is the isotropic pressure.

**Figure 4:**
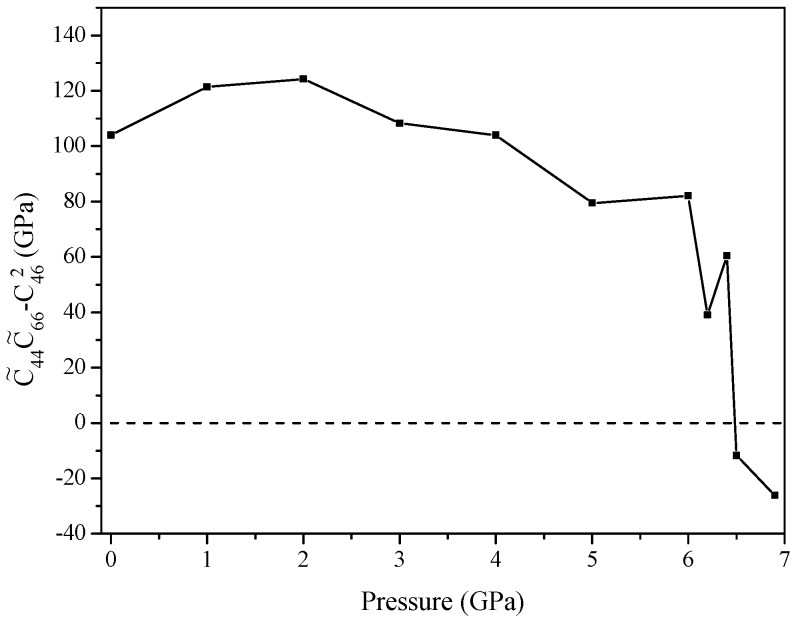
Calculated ${\tilde{C}}_{44}{\tilde{C}}_{66}-C_{46}^{2}$ of *C*2/*m*-Ca_2_C under different pressures.

**Figure 5:**
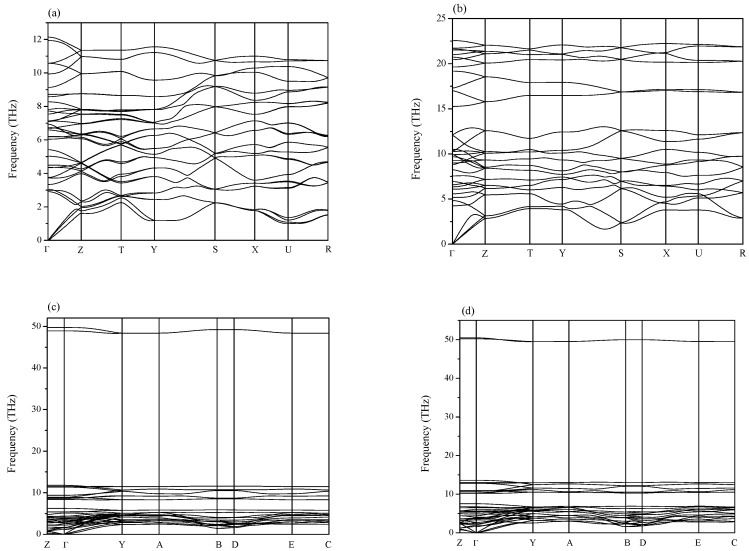
Phonon spectra for (**a**) *Pnma*-Ca_2_C at 0 GPa; (**b**) *Pnma*-Ca_2_C at 100 GPa; (**c**) *C*2/*m*-Ca_2_C at 0 GPa; (**d**) *C*2/*m*-Ca_2_C at 6.4 GPa.

**Figure 6:**
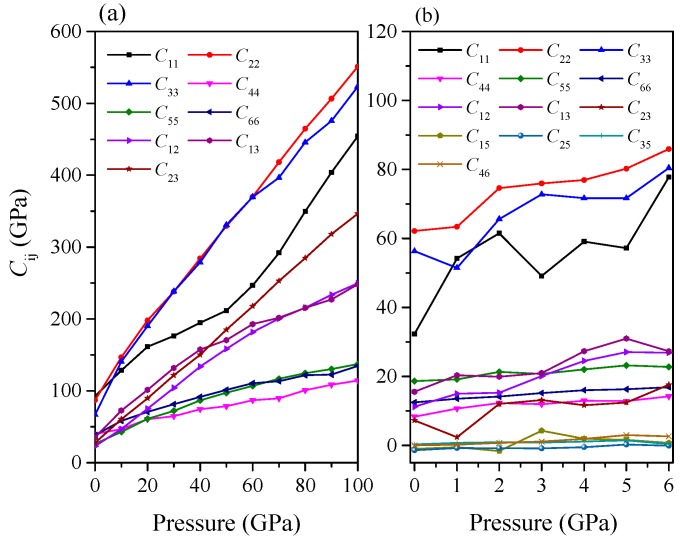
Elastic constants as a function of pressure. (**a**) *Pnma*-Ca_2_C; (**b**) *C*2/*m*-Ca_2_C.

If the elastic constants satisfy these stability criteria, it means the structure is mechanically stable. From Table 3, one can see that orthorhombic *Pnma*-Ca_2_C is mechanical stable up to at least 100 GPa. For monoclinic *C*2/*m*-Ca_2_C, the criteria ${\tilde{C}}_{44}{\tilde{C}}_{66}-C_{46}^{2}>0$, which is similar to the Equation (9), is obeyed only up to 6.4 GPa, as seen in Figure 4, showing that it has mechanical stability below 6.4 GPa. Furthermore, the phonon spectra are presented in Figure 5 to ensure the dynamical stability. As observed, there is no imaginary frequency in the whole Brillouin zone, indicating that *Pnma*-Ca_2_C is dynamically stable up to at least 100 GPa and that the *C*2/*m*-Ca_2_C is dynamically stable below 6.4 GPa. The elastic constants as a function of pressure are displayed in Figure 6 with an approximately upward tendency. We noticed that, for *Pnma*-Ca_2_C, *C*_11_ is larger than *C*_22_ or *C*_33_ at 0 GPa, whereas it is less than *C*_22_ or *C*_33_ at high pressures, which is in consistent with our previous analyses on the incompressibility along the *a*-, *b*-, and *c*-axis.

In Table 3, the bulk modulus *B* and shear modulus *G* are calculated by Voigt-Reuss-Hill approximations [20,21,22]. The Young’s modulus *E* and Poisson’s ratio, υ are given by the following equations [22]:

(16) $$E=\frac{9BG}{3B+G},\upsilon =\frac{3B-2G}{2\left(3B+G\right)}$$

The *Pnma*-Ca_2_C is larger than *C*2/*m*-Ca_2_C in bulk modulus, shear modulus and Young’s modulus at 0 GPa, as listed in Table 3. All the elastic modulus increase with pressure for *Pnma*-Ca_2_C. According to Pugh [23], the brittle material has a small *B*/*G* ratio (*B*/*G* < 1.75), whereas, the ductile material has a larger ratio (*B*/*G* > 1.75). It is interesting that *Pnma*-Ca_2_C and *C*2/*m*-Ca_2_C show the brittle manner at 0 GPa and transform to ductile manner at 9.3 GPa and 2.0 GPa, respectively.

Calculating the elastic anisotropy of crystal is of great importance to further study the physical and chemical properties. The calculated universal elastic anisotropy index (*A^U^*), shear anisotropic factors (*A*_1_, *A*_2_ and *A*_3_) and percentage of anisotropy in compressibility and shear (*A_B_* and *A_G_*) are listed in Table 4. For arbitrary symmetry, the universal elastic anisotropy index *A^U^* is obtained by [24,25]:

(17) $$A^{U}=5\frac{G_{V}}{G_{R}}+\frac{B_{V}}{B_{R}}-6$$

When *A^U^* is 0, it means the solid is isotropic, otherwise the solid is anisotropic. The results of *Pnma*-Ca_2_C are 0.37 at 0 GPa, 0.62 at 50 GPa and 0.15 at 100 GPa, respectively. And the results of *C*2/*m*-CaC_2_ are 0.95 at 0 GPa and 0.54 at 6 GPa, respectively. All of them are larger than 0, indicating an elastic anisotropy. The shear anisotropic factors provide a measure of the degree of anisotropy in the bonding between atoms in different planes. The shear anisotropic factor for the {100} shear plane between the <011> and <010> directions is [26,27]:

(18) $$A_{1}=\frac{4C_{44}}{C_{11}+C_{33}-2C_{13}}$$

For the {010} shear plane between the <101> and <001> directions, it is:

(19) $$A_{2}=\frac{4C_{55}}{C_{22}+C_{33}-2C_{23}}$$

For the {001} shear plane between the <110> and <010> directions, it is:

(20) $$A_{3}=\frac{4C_{66}}{C_{11}+C_{22}-2C_{12}}$$

The factors *A*_1_, *A*_2_ and *A*_3_ are 1.0 for any isotropic crystals. As observed in Table 3, all the calculated shear anisotropic factors are not 1.0, presenting the elastic anisotropy. The percentage anisotropy in compressibility and shear are defined as [26]:

(21) $$A_{B}=\frac{B_{V}-B_{R}}{B_{V}+B_{R}},$$

(22) $$A_{G}=\frac{G_{V}-G_{R}}{G_{V}+G_{R}},$$

where *B* and *G* are the bulk and shear modulus, and the subscripts *V* and *R* represent the Voigt and Reuss bounds. The values of isotropic crystal are 0.0. In Table 3, the values of *A_B_* and *A_G_* suggest that these two structures of Ca_2_C are anisotropic in compressibility and shear.

**Figure 7:**
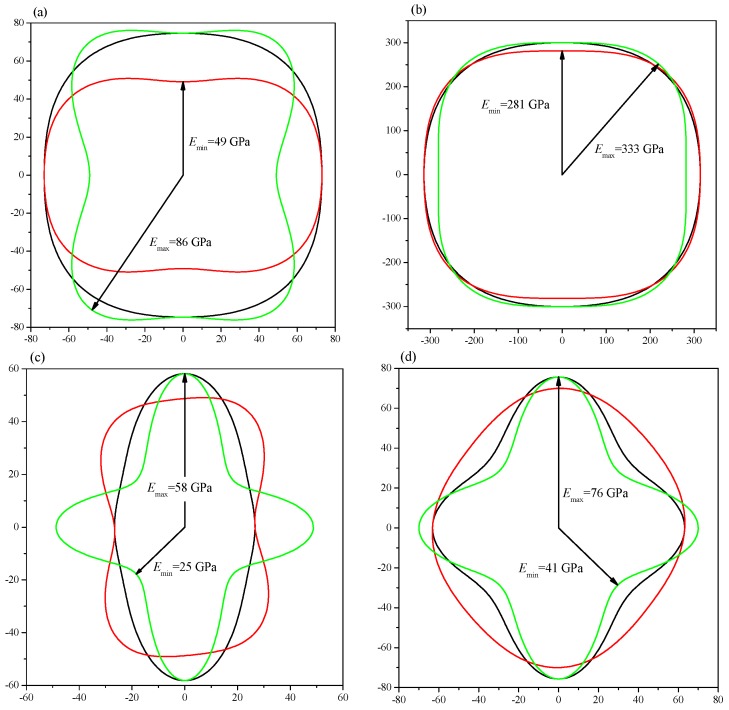
2D representations of the Young’s modulus. (**a**) *Pnma*-Ca_2_C at 0 GPa; (**b**) *Pnma*-Ca_2_C at 100 GPa; (**c**) *C*2/*m*-Ca_2_C at 0 and 6 GPa; (**d**) *C*2/*m*-Ca_2_C at 6 GPa. The black, red and green lines represent the *xy*, *xz* and *yz* planes, respectively.

**Figure 8:**
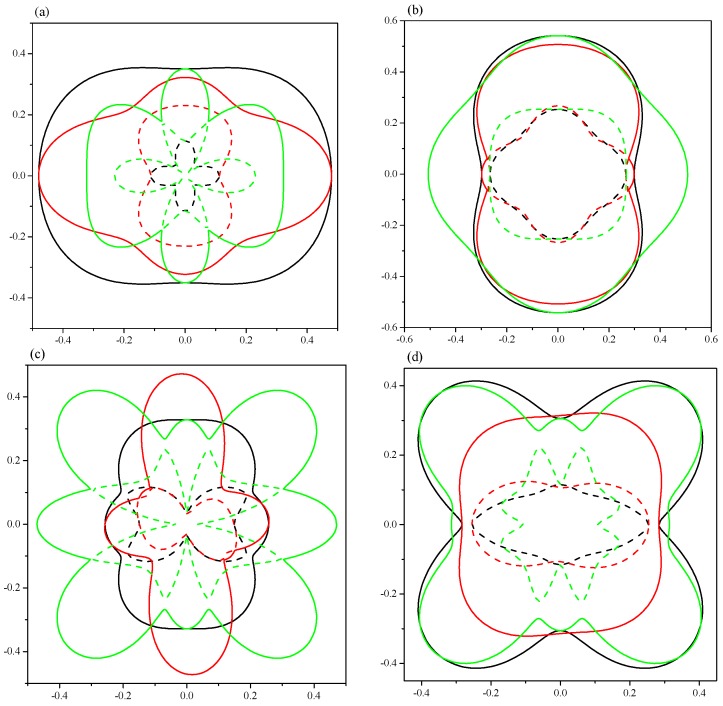
2D representations of Poisson’s ratio. (**a**) *Pnma*-Ca_2_C at 0 GPa; (**b**) *Pnma*-Ca_2_C at 100 GPa; (**c**) *C*2/*m*-Ca_2_C at 0 GPa; (**d**) *C*2/*m*-Ca_2_C at 6 GPa. The solid and dash lines represent the maximal and minimal positive values, respectively. The black, red and green lines represent the *xy*, *xz* and *yz* planes, respectively.

**Figure 9:**
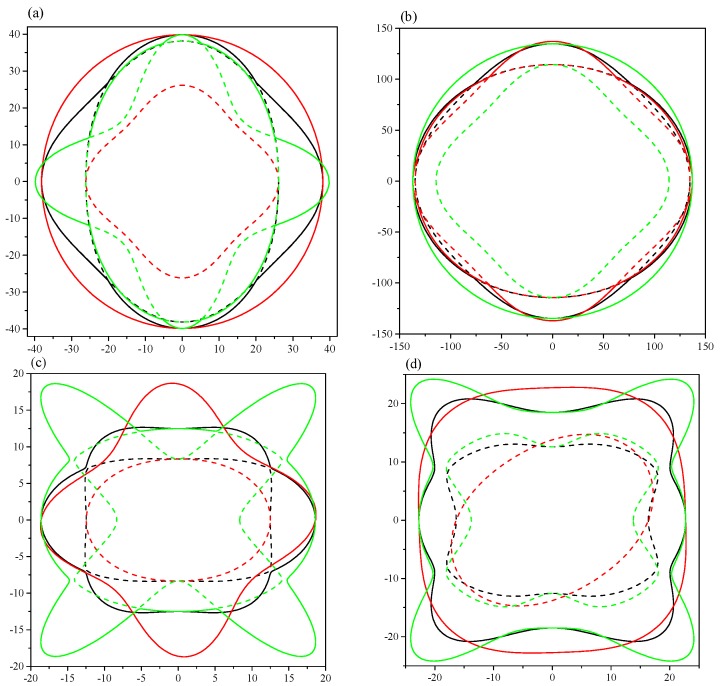
2D representations of shear modulus. (**a**) *Pnma*-Ca_2_C at 0 GPa; (**b**) *Pnma*-Ca_2_C at 100 GPa; (**c**) *C*2/*m*-Ca_2_C at 0 GPa; (**d**) *C*2/*m*-Ca_2_C at 6 GPa. The solid and dash lines represent the maximal and minimal positive values, respectively. The black, red and green lines represent the *xy*, *xz* and *yz* planes, respectively.

To intuitively illustrate the elastic anisotropy, the directional dependence of elastic anisotropy was calculated by the ELAM code [28], which shows the 2D figures of the differences in each direction. The calculated Young’s modulus along different directions as well as the projections in different planes are demonstrated in Figure 7. The ratios of *E*_max_/*E*_min_ are 1.76 (1.19) and 2.32 (1.85) for *Pnma*-Ca_2_C at 0 (100) GPa and *C*2/*m*-Ca_2_C at 0 (6.0) GPa, respectively, which means *C*2/*m*-Ca_2_C has greater anisotropy. The anisotropy in *yz* plane is the greatest for *Pnma*-Ca_2_C at both 0 and 100 GPa (see Figure 7a,b). In Figure 7c,d, the *C*2/*m*-Ca_2_C also has the largest anisotropy in *yz* plane at both 0 and 6 GPa. The 2D representations of Poisson’s ratio are revealed in Figure 8. All of them show the elastic anisotropy. From Figure 8a,b, it can be found that the *Pnma*-Ca_2_C has the greatest anisotropy in *yz* plane at 0 GPa and in *xz* plane at 100 GPa. However, the greatest anisotropy of *C*2/*m*-Ca_2_C is in *yz* plane at both 0 and 6 GPa (see Figure 8c,d). The *C*2/*m*-Ca_2_C is more anisotropic than the *Pnma*-Ca_2_C in Poisson’s ratio. As far as the 2D projections of shear modulus in *xy*, *yz*, and *xz* planes shown in Figure 9, both *C*2/*m*-Ca_2_C and *Pnma*-Ca_2_C exhibit the obvious elastic anisotropy. From Figure 9a,b, it is seen that the 2D projections of shear modulus in *xz* plane at 0 GPa and in *yz* plane at 100 GPa are almost a perfect circle, showing a slight anisotropy character in these two cases. The anisotropy of *Pnma*-Ca_2_C at high pressure is smaller than that at 0 GPa. The same case occurred for *C*2/*m*-Ca_2_C, as seen in Figure 9c,d. Similar to the anisotropy of Poisson’s ratio, the shear modulus of *Pnma*-Ca_2_C has the greatest anisotropy in *yz* plane at 0 GPa and in *xz* plane at 100 GPa, and that of *C*2/*m*-Ca_2_C is the most anisotropic in *yz* plane at both 0 and 6 GPa.

**Figure 10:**
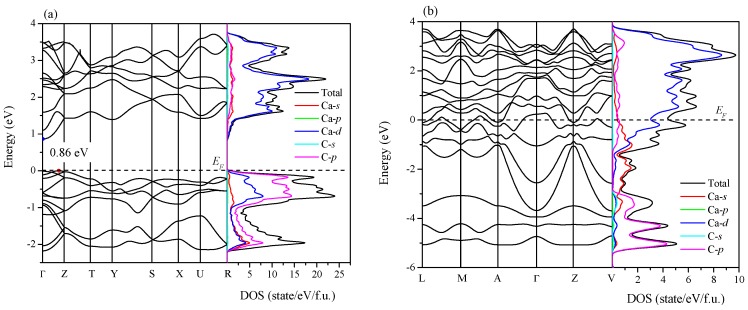
Electronic band structure and density of state of *Pnma*-Ca_2_C (**a**) and *C*2/*m*-Ca_2_C (**b**) at 0 GPa.

As shown in Figure 10, a research of the electronic band structure and density of state (DOS) of Ca_2_C at 0 GPa was also made. The dashed line represents the Fermi level (*E_F_*). From Figure 10a, one can see that *Pnma*-Ca_2_C is a semiconductor characterized by a quasi-direct band gap of 0.86 eV (the direct band gap at Γ point is 0.87 eV). The conduction band minimum (CBM) is just at Γ point, and the valence band maximum (VBM) locates at (0, 0, 0.378) along the Γ-Z direction. The calculated band gap of *Pnma*-Ca_2_C at 14 GPa is direct band gap with 0.65 eV, which is close to the previous value of 0.64 eV [9]. It is known that the calculated band gap with DFT is usually underestimated by 30%–50%, so the ideal band gap is larger than this calculated result. The DOS of *Pnma*-Ca_2_C near Fermi level is mainly originated from the contributions of C-*p* orbital electrons. In Figure 10b, the calculated electronic band structure crosses the Fermi level along many directions in the Brillouin zone, showing the metallic character. And the DOS near Fermi level is mainly characterized by the Ca-*d* orbital electrons.

## 3. Computational Methods

Our calculations are performed via the generalized gradient approximation (GGA) parameterized by Perdew-Burke-Eruzerhof (PBE) [29] in the Cambridge Serial Total Energy Package (CASTEP) code [30], which is based on the density functional theory (DFT) [31,32]. For the two Ca_2_C phases, the ultrasoft pseudo-potential [33] which describes the interactions between the ionic core and valence electrons is used with the cutoff energy of 420 eV. The k-points of Pnma-Ca_2_C (7 × 11 × 6) and *C*2/*m*-Ca_2_C (6 × 9 × 3) in the first irreducible Brillouin zone are generated using Monkhorst-Pack mesh scheme [34]. Furthermore, the Broyden-Fletcher-Goldfarb-Shanno (BFGS) minimization scheme [35] is used in geometry optimization. The convergence is within 1 meV/atom in the total energy convergence tests for all calculation parameters. The self-consistent convergence of the total energy is 5 × 10^−6^ eV/atom, the maximum force on the atom is 0.01 eV/Å, the maximum stress is 0.02 GPa and the maximum ionic displacement is 5 × 10^−4^ Å. 

## 4. Conclusions

A systematic analysis of the pressure-induced phase transition of Ca_2_C is made by first-principles calculations. The enthalpy and dependence of volume on pressure of Ca_2_C are performed. We found that there is a phase transition which occurs at 7.8 GPa transforming from *C*2/*m*-Ca_2_C to *Pnma*-Ca_2_C with a volume drop of 26.7%. The *Pnma*-Ca_2_C is larger than *C*2/*m*-Ca_2_C in the calculated bulk modulus, shear modulus, Young’s modulus and Poisson’s ratio at 0 GPa. Both of them exhibit the elastic anisotropy. The low-pressure phase *C*2/*m*-Ca_2_C, which is mechanically stable up to 6.4 GPa, has the greater anisotropy over the *Pnma*-Ca_2_C. The electronic band structures reveal the semiconductivity of *Pnma*-Ca_2_C and the metallicity of *C*2/*m*-Ca_2_C. The quasi-direct band gap of *Pnma*-Ca_2_C at 0 GPa is 0.86 eV. Furthermore, the total and partial density of states is provided to study the specific contribution to Fermi level.

## Figures and Tables

**Table 1:** Bader charge analysis for *C*2/*m*- and *Pnma*-Ca_2_C at 7.8 GPa.

Phase	Ionic Radius (Å)		Charge Transfers (*e*)
	C	Ca	Ca → C
*C*2/*m*	1.534	1.871	0.928
*Pnma*	1.788	1.485	2.348

**Table 2:** Lattice parameters of Ca_2_C at various pressures.

Phase	Pressure (GPa)	*a* (Å)		*b* (Å)		*c* (Å)		*β* (deg)		*V* (Å^3^)	
*Pnma*	0	6.677	6.689 ^a^	4.384	4.389 ^a^	7.979	7.981 ^a^			233.58	234.32 ^a^
	10	6.404	6.415 ^a^	4.150	4.154 ^a^	7.513	7.518 ^a^			199.68	200.35 ^a^
		6.449 ^b^		4.157 ^b^		7.523 ^b^				201.7 ^b^	
	30	5.919	5.929 ^a^	3.931	3.933 ^a^	7.195	7.204 ^a^			167.41	168.04 ^a^
*C*2/*m*	0	7.166		3.775		15.490		122.9		351.59	
	5	6.743	6.701 ^a^	3.594	3.587 ^a^	14.65	14.68 ^a^	122.8	122 ^a^	298.32	
	6.4	6.674		3.554		14.45		122.6		288.87	

^a^ Calculated data in Ref. [9]; ^b^ Experimental results in Ref. [9].

**Table 3:** Calculated elastic constants *C*_ij_ (GPa), bulk modulus *B* (GPa), shear modulus *G* (GPa), Young’s modulus *E* (GPa), Poisson’s ratio υ, and *B*/*G* ratio of Ca_2_C at various pressures.

Pressure (GPa)	*Pnma*-Ca_2_C			*C*2/*m*-Ca_2_C		
	0	50	100	0	6	6.4
*C*_11_	92	212	454	32	78	71
*C*_22_	87	329	551	62	86	84
*C*_33_	67	331	523	56	80	86
*C*_44_	40	79	114	8	13	14
*C*_55_	26	97	137	19	23	20
*C*_66_	38	101	135	13	17	15
*C*_12_	23	158	250	11	26	28
*C*_13_	35	170	248	16	27	29
*C*_23_	27	185	347	7	18	23
*C*_15_				−1	0.75	4
*C*_25_				−1.4	−0.06	3
*C*_35_				0.3	0.44	−1.31
*C*_46_				0.04	2.65	2.67
*B*	46	203	353	24	43	45
*G*	30	75	122	15	21	19
*E*	74	200	328	37	54	50
υ	0.23	0.34	0.35	0.24	0.29	0.31
*B*/*G*	1.53	2.71	2.89	1.60	2.05	2.37

**Table 4:** Calculated universal elastic anisotropy index *A^U^*, shear anisotropic factors *A*_1_, *A*_2_ and *A*_3_, and percentage of anisotropy in compressibility and shear *A_B_* and *A_G_* (in %) of Ca_2_C.

Phase	Pressure (GPa)	*A^U^*	*A*_1_	*A*_2_	*A*_3_	*A_B_*	*A_G_*
*Pnma*	0	0.37	1.79	1.06	1.14	0.6	3.5
	50	0.62	1.56	1.34	1.81	3.8	5.1
	100	0.15	0.95	1.44	1.06	1.3	1.2
*C*2/*m*	0	0.95	0.58	0.72	0.69	2.7	8.2
	6	0.54	0.53	0.70	0.62	0.06	5.1
